# Experimental and computational studies of cellulases as bioethanol enzymes

**DOI:** 10.1080/21655979.2022.2085541

**Published:** 2022-06-22

**Authors:** Shrivaishnavi Ranganathan, Sankar Mahesh, Sruthi Suresh, Ayshwarya Nagarajan, Taner Z. Sen, Ragothaman M.Yennamalli

**Affiliations:** a Department of Biotechnology, School of Chemical and Biotechnology, SASTRA Deemed to be University, Tirumalaisamudram, Thanjavur, India; b Department of Bioinformatics, School of Chemical and Biotechnology, SASTRA Deemed to be University, Tirumalaisamudram, Thanjavur, India; c U.S. Department of Agriculture, Agricultural Research Service, Crop Improvement and Genetics Research Unit, 800 Buchanan Street, Albany, California 94710, United States of America

**Keywords:** Cellulase, pretreatment process, property evaluation, computational methods, biofuel

## Abstract

Bioethanol industries and bioprocesses have many challenges that constantly impede commercialization of the end product. One of the bottlenecks in the bioethanol industry is the challenge of discovering highly efficient catalysts that can improve biomass conversion. The current promising bioethanol conversion catalysts are microorganism-based cellulolytic enzymes, but lack optimization for high bioethanol conversion, due to biological and other factors. A better understanding of molecular underpinnings of cellulolytic enzyme mechanisms and significant ways to improve them can accelerate the bioethanol commercial production process. In order to do this, experimental methods are the primary choice to evaluate and characterize cellulase’s properties, but they are time-consuming and expensive. A time-saving, complementary approach involves computational methods that evaluate the same properties and improves our atomistic-level understanding of enzymatic mechanism of action. Theoretical methods in many cases have proposed research routes for subsequent experimental testing and validation, reducing the overall research cost. Having a plethora of tools to evaluate cellulases and the yield of the enzymatic process will aid in planning more optimized experimental setups. Thus, there is a need to connect the computational evaluation methods with the experimental methods to overcome the bottlenecks in the bioethanol industry. This review discusses various experimental and computational methods and their use in evaluating the multiple properties of cellulases.

## Graphical_Abstract

**Figure:**
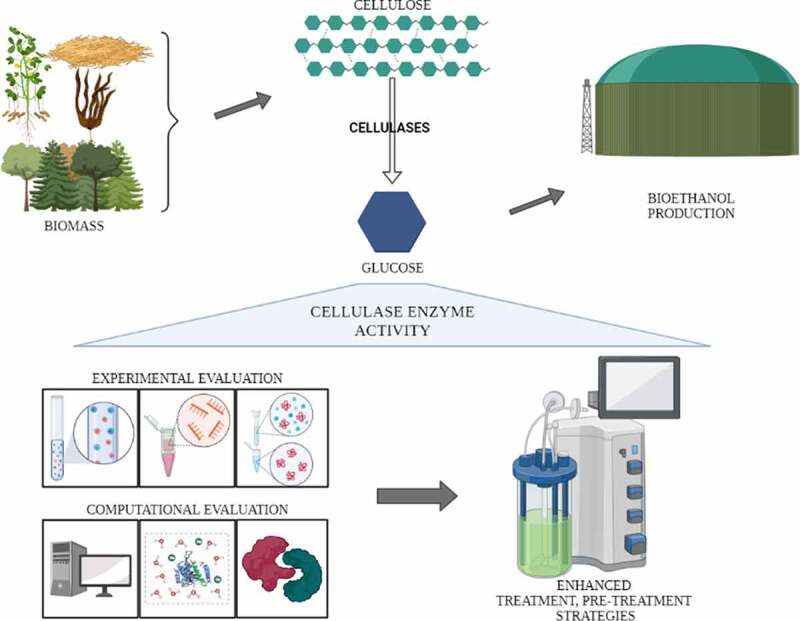

## Research_Highlights

- Methods and tools to evaluate cellulases can improve bioethanol production.
- Cellulases enzyme mechanisms are usually studied using experimental techniques.
- Computational evaluation of cellulases’ properties reduces cost and time.
- Combination of different evaluation methods can aid in optimization of cellulases and the glucose yield.

## 1. Introduction

The United Nations (https://www.un.org/en/sections/issues-depth/population/index.html) predicts the increase of the world population by 2 billion persons in the next 30 years, i.e., the current population of 7.7 billion may reach 9.7 billion by 2050. Energy sources are at the most significant threat because of their versatile use in human development. Necessary human activities like mobility, health, communication, irrigation, cooking, space travel, etc., are at the cost of depleting energy sources [1,2]. The answer to this critical question of the increasing energy demand is currently met, in large, by fossil fuels. It is a known fact that natural resources and renewable energy improve environmental quality in the distant future [3]. The transition from fossil fuels to renewable sources of energy and new advances in energy storage systems opens the possibility of clean fuel and an opportunity to tackle climate change [4–8]. Biofuels are one of the options for mitigating dependency on fossil fuels and reducing carbon emissions in the global energy system [9].

### 1.1. Generation of biofuels

There are three generations of biofuels: namely, first-, second-, and third-generation biofuels, where the categorization is based on 1) the biomass sources used, 2) the limitations of these biomass sources, and 3) their technological progress [10,11].

The first-generation biofuels come from edible biomass or food crops like corn, sugar beets, sugarcane, wheat, grains, industrial sweet potatoes, oilseeds, vegetable oils, and rendered animal fats. The second-generation biofuels come from non-edible biomass such as wood, sawdust, wheat straws, corn husks, seed waste, manure, paper waste, household waste, wastewater, etc. [12,13]. In the last decade, researchers have been focusing on second-generation biomass for biofuel. Research focus in the recent decade has been on, but not limited to, wood bark [14], olive stone [15], pine pellets [16], avocado stone [17], wheat straw, wood [18], walnut shell [19], peanut shell [20], mango stone [21], sunflower seed husk [22], corn cob waste [23], palm oil kernel shell [24], and others. The third-generation biomass for biofuels like algae [25] and woody biomass are considered a better alternative than second-generation because they do not compete with food/feed sources. However, they are limited by economic feasibility because of the high cost of production and treatments [26,27].

Compared to first- and third-generation biomass, the second-generation biomass is relatively more sustainable [28]. This is because they are the byproducts of agricultural industry and there is no additional requirement of land, water, and fertilizer use to derive these sources. The agricultural plant wastes are majorly lignocellulosic biomass, composed of lignin, cellulose, and hemicellulose that constitute the plant cell wall, where the recalcitrant polysaccharides and lignin are strongly cross-linked via ester and ether linkages [29–31].

### 1.2. Pretreatment of biomass

**Figure 1.**
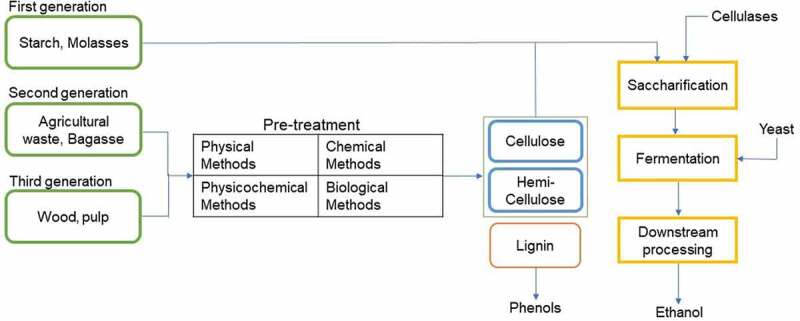
**Biomass to bioethanol: schematic representation of the overall bioconversion process**. while first generation biomass is mainly starch and sugarcane-derived polymers, second and third generation biomass are grasses, agricultural wastes, feedstock, and genetically modified plants with reduced lignin and hemicelluloses content. Multiple methods (physical, chemical, physicochemical, and biological) can be used to breakdown the cell wall components in the upstream of bioethanol conversion. *Images used from www.creativecommons.org under creative commons license for reproducing.*

Regardless of first-, second-, or third-generation biomass, pretreatment is a required process for the biomass to be utilized to its full potential. Pretreatment is the process to weaken and break these strong cross-links, so that the recalcitrant polymers are amenable to hydrolysis with cellulases into simpler sugars [32]. The general biomass pretreatment process is shown in Figure 1. There are many types of pretreatments, and they are categorized into: 1) physical pretreatment processes that include milling, irradiation, extrusion, pyrolysis, etc.; 2) chemical pretreatment processes that include acid treatment, alkali treatment, use of ionic liquids, organosolv, etc.; 3) physico-chemical pretreatment processes that include steam explosion, liquid hot water, ammonia fiber explosion, ammonia recycling percolation, wet oxidation, etc.; and 4) biological pretreatment processes using an enzyme cocktail. All these pretreatment methods loosen up the cellulose fibers and further degradation by the cocktail of enzymes leads to the release of glucose, which releases ethanol after fermentation. The pretreatment step is essential for removing some by-products that inhibit enzyme activity [33,34]. These by-products bind to the enzyme’s active site or cavity and prevent the turnover of the enzyme for subsequent reactions. There are multiple reports of hybrid pretreatment methods, where a combination of physical, chemical, and biological methods have been used. Table 1 lists the various types of pretreatment processes individually with their advantages and disadvantages.

**Table 1.** Various lignocellulose pretreatment process, their process conditions, advantages and disadvantages.

Pretreatment methods		Process conditions	Advantages	Disadvantages	Reference
**Physical methods**					
	Disk milling	Milling (10–30 mm) and grinding, particle size (0.2–2 mm)	No need of chemical, it is scalable	It is highly energy intensive process, poor in sugar conversion	[35]
	Extrusion	Screw speed, 350 rpm, barrel temperature, 80 °C, 40 % moisture.	Low pretreatment temperature and degradation products not formed, no need of washing, can be used continuously.	High energy cost, needs more aberration of metal surface.	[36]
	Microwave radiation	Microwave 680 W, irradiation time 24 min and substrate concentration 75 g/L.	Less processing time, less energy input than conventional heating, and high uniformity and selectivity.	Reactor cost is high, needed additional safety, sugar conversion and substrate concentration are low.	[37]
	Pyrolysis	1 N sulfuric acid, temperature at 97 °C for 2.5 hours.	More efficient when carried out in the presence of oxygen at low temperature.	Loge solid residence time.	[38]
**Chemical methods**					
(1) Acid pretreatment	Dilute sulfuric acid	Temperature 140–190 °C, 0.4–2 % sulfuric acid, resident time 1–40 min.	Used for wide range of biomass, and during pretreatment process produce hydrolyzed xylose.	Need to use costly hastelloy reactor, controlling reaction condition is not easy, produces toxic degradation and during recycling water removal of salt is costly.	[39]
	Organic acid	Temperature 130–190 °C, 50–90 mM of organic acid.	Fractionation of biomass into soluble lignin rich hemicellulose stream, and low reaction pressure.	More water needed to clean substrate after pretreatment and acid recovery is very costly.	[40]
	Concentrated acid	Shorter residence time.	In some case no need of enzyme for cellulose depolymerization, cellulose is converted to well reactive amorphous cellulose when phosphoric acid is used. It is very effective on softwood.	The step of acid recovery is energy exhaustive.	[41]
	Acidic organosolv	Acetone-water pretreatment (acetone : water molar ratio of 1 : 1) at temperature 195 °C, pH 2.0, and residence time 5 minutes.	It can separate pure lignin stream, removal of lignin enhance the digestibility of cellulose.	High-pressure operation has high risk and used solvents are flammable and volatile.	[42]
	SPORL	Temperature 180 °C, residence time 25 minutes and ratio of liquor/wood = 3 : 1 v/w.	Removal of lignin is more effective and high sugar yields, recovered components of biomass in less chemical transformed forms.	The degradation of sugar at harsh conditions, post pretreatment process used large water and pretreatment chemical recovery is very costly.	[43]
(2) Neutral pretreatment	Ionic liquid	Temperature 100–150 °C and residence time few minutes to hour.	Carbohydrate losses are low and only at severe condition , degradation products are significant.	Solvent loading, solvent cost and cost of solvent regeneration are very high.	[44]
	Liquid hot water	Temperature 160–220 °C, 15 minutes residence time.	No need of external chemical, and reactor system is simple.	Use of more water, loss of some hemicelluloses in water stream and loading of solids is low.	[44]
	Ozonolysis	Room temperature, Ozone sparging.	Lignin removal is effective, the production of inhibitory products is very low and reaction can be performed at atmospheric conditions.	Large amount of ozone is required i.e., costly and some portion of lignin is lost during pretreatment process.	[45]
(3) Alkaline Pretreatment	Ammonia Fiber Explosion (AFEX)	Temperature 100–140 °C, 1 : 1–2 : 1 ammonia to biomass loading, residence time 30–60 minutes, 60–100 % moisture.	Volatile ammonia can be recovered and reused, degradation product form very less and lignin is relocated on the surface that help to densify the biomass.	Safety issues in use of ammonia, recovery of ammonia is costly and not proficient for hardwood biomass.	[46]
	Ammonia recycled percolation (ARP)	Temperature 160–180 °C, 10–30 minutes residence time, 0.5 gm ammonium hydroxide per gm of biomass.	Removal of recalcitrant lignin efficiently and it works very good for grasses.	Use of high amount of water, energy exhaustive process, and not effective for hardwood biomass.	[47]
	Soaking in aqueous ammonia (SAA)	Solid to liquid ratio 1 : 11, temperature 60 °C, and residence time 8–24 hours.	Lower reaction temperature needed.	Residence time is very long, use of large water and scale-up issues.	[48]
	NaOH	-	Highly reactive cellulose conversion and solubilization of lignin.	High residence time, use of large water, scale-up issues and recovery of catalyst is costly.	[49]
	Alkaline H_2_O_2_	0.5–2 % sodium hydroxide, 0.125 g H_2_O_2_/g biomass, temperature 22 °C, and atmospheric pressure for 48 hours.	Milder pretreatment condition, scalable and commercially used in paper industry.	Use of large water, expensive catalytic recovery, and due to oxidation process energy content of lignin is lost.	[50]
	Lime	Temperature 25–160 °C, residence time 120 minutes to weeks, 0.07–0.2 g CaO/g biomass.	Pretreatment can be done using Inexpensive pretreatment reactor system.	Requirement of large water, expensive catalytic recovery and long residence time.	[51]
	Alkaline wet oxidation	Temperature >120 °C, 0.5 2Mpa, <30 minutes residence time.	Dry to dry process and formation of lesser degradation products.	Need of high pressure equipment, high cost of oxygen that is used as a catalyst, and oxidation of lignin makes it lesser dense in energy.	[52]
**Physiochemical methods**					
	Steam explosion	Temperature 180–210 °C, 1–120 minutes residence time and 0.7–4.8 MPa pressure.	Works effectively both for hardwood and herbaceous biomass.	Expensive reactor system requirement due to high pressure operation.	[53]
	Supercritical CO_2_	Temperature 112–165 °C, 0–73 % moisture, 10–60 minutes residence time and pressure 1000–4000 psi.	Less corrosive, nontoxic chemical, non-flammability, and stream not wasted.	Need of high pressure reactions, and need of expensive reactor system which can tolerate high pressure.	[54]
	Oxidative	Temperature >120 °C for 30 minutes residence time.	Oxygen and alkali addition to the wet oxidation process reduces the severity of the medium and inhibitors formation. Ozonolysis forms a negligible amount of inhibitors.	Solvents need to be separated, recovered and reused as they have high cost. Needs washing step.	[52]
**Biological method**					
		Temperature 25–30 °C, solid state fermentation, 80–120 % moisture, and 10–15 days residence time.	The pretreatment is selective, requires no chemicals addition, uses less energy and has low severity. It is an environmentally friendly process.	Enzymatic hydrolysis has long incubation time, low production rate and high sensitivity to inhibition. Loss of cell activity requires high control conditions.	[55,56]

### 1.3. Cellulases and their importance in biofuel production

The hydrolysis of cellulose is a complex process that involves the interaction of cellulase enzyme with multiple cellulose chains. The increased hydrolysis of the cellulose chains results in a higher yield of glucose from cellulose. Cellulases are potential modular enzymes (discrete units in a multi domain protein, where the functions are separable [57–60]) hydrolyzing insoluble cellulose to soluble oligosaccharides. Cellulases are important biofuel enzymes because of their ability to hydrolyze cellulose into glucose, a sugar that can be fermented to ethanol. Cellulases, similar to any enzyme, are affected by numerous external parameters that in turn cause changes in their activity. Parameters such as pH, temperature, substrate concentration, etc. affect the structural stability, enzymatic activity, and ultimately the glucose yield.

### 1.4. Latest advances of cellulases for biofuels and biorefinery

Extraction of ethanol from biomass is achieved by techniques of biorefinery, and there are several methods to do it [61], and over the course of time, enzymatic refining procedures have proven to be the most economic, and also give the best yield. In these practices, the usage of cellulases for ethanol production from lignocellulosic biomass is quite familiar, but this method faces issues: slower conversion rates due to biomass retention or recalcitrance [62], high cost and scale-up challenges [63]*. Various improvement strategies have been explored in this regard [64]. The simplest approach is to employ a synergistic cocktail of enzymes as accessory enzymes to complement cellulases, such as xylanases and lytic polysaccharide monooxygenases [64], and cellobiohydrolases and endoglucanases [65]. Co-expression of cellulase and xylanase enzyme genes in *Saccharomyces cerevisiae* led to efficient hydrolysis of LCB, better than wild-type *S.* *cerevisiae* [66]. Wang et al. proved the involvement of extracellular products of white-rot fungus in enhancing cellulase function [67].

Hot water pre-treatments have been tested for promoting autohydrolysis before complete hydrolysis of biomass [68]. However, the effects of this step on cellulases are inconclusive, and more research in this area is required. An integrated process employed by Lian et al. [69], where autohydrolysis, nanofiltration and xylanase hydrolysis are combined to give a prebiotic that is processed better than traditional multi-process techniques is an attractive novel approach. An alternative is to engineer proteins by inducing deliberate mutations, seeking structure–function relationships, to give suitable results [70].

### 1.5. Enzyme mimicking nanomaterials

Current technologies focusing on lignin degradation are expensive, leave undesirable and wasteful residues (whose disposal incurs additional costs), and sometimes can cause formation of unwanted compounds. To address these setbacks, greener methods of lignin depolymerization are being approached. Specifically, nanomaterial-based enzymes have been approached for their inherent enzyme-like properties and increased surface area to volume ratio, improving reaction rates. Deng et al., explored the usage of palladium nanoparticles supported by cerium oxide [71]. Another study employed Nickel nanoparticles to get a better yield of saturated hydrocarbons after performing a special type of chemical extraction called the organosolv process [72]. Molybdenum oxide supported by carbon nanotubes were deemed as an economic alternative to reduce lignin to phenolic derivatives which prove useful for further processes of biofuel production [73]. A Fenton-like process utilizing iron oxide nanoparticles by mimicking their peroxidase activity to reduce lignin was successful in the process while also not detrimentally impacting the carbohydrate content of the biomass [74]. This approach is gaining research limelight, and many versions and derivatives are under investigation. Several advantages provided by nanomaterial-based enzymes are enhanced reaction kinetics, low mass transfer resistance, better flexibility of reactor design, assured recovery which prompts reuse, thereby becoming more economic, and stability in various reaction conditions [75]. These advantages have promoted nanozyme-based biofuel cell research in recent times.

### 1.6. Effect of substrates produced in pretreatment

Pretreatment of lignocellulosic biomass has become a pre-requisite during the process of biofuel production, which helps in superior cellulase-mediated catalysis [76]. While physical and chemical procedures for the same have their places, they tend to have harsh impacts on reactor walls and/or reactor constituents. Hydrothermal methods are a suitable alternative in this regard. Currently, their application has spread to many operations in the lignocellulosic biomass biorefinery set of procedures. It gives the liberty of enabling flexible temperature and pressure setting based on the intention of the process, with two main kinds of methodologies: subcritical and supercritical operations, with reference to the critical point of water. Many types of reactors (batch, semi-continuous, continuous, and integrated) have been employed for the hydrothermal treatment of various types of biomass, but full-fledged commercial scale operations are yet to be implemented. More interest in this area is underway, and their results will help in finding a feasible approach for lignocellulosic biomass pretreatment by hydrothermal techniques like steam explosion.

The use of improved strain of *Trichoderma reesei* RUT-C30, which has β-glucosidase gene from *Talaromyces emersonii* and invertase gene from *Aspergillus niger* heterologously expressed, has improved the yield of glucose by 50 % [77]. In contrast, the ionic liquid method yields 81.5 % ethanol conversion, but the downside is the high ionic liquid cost [78]. Recent reviews support the novel and multiple pretreatments optimization of lignocellulose biomass, including greener pretreatment technologies [79–81].

### 1.7. Reactor design

Enzymatic degradation of lignocellulosic biomass in a large-scale bioreactor is the rate-limiting step for biofuel production because it incurs a higher cost, and the prospects of enzyme inhibitors and undesirable intermediates are significant. Therefore, the design of the bioreactor plays a pivotal role in addressing these issues [82]. The problem of the enzyme being expensive is approached by the recycling of cellulases in the reactor. This is accomplished by various methods such as recycling in the liquid or solid medium, readsorption into fresh medium, whole slurry recycling technique, membrane retention followed by concentration, and enzyme immobilization [83]. Processing higher amount of biomass may seem like a tempting option to consider, but slurries above 20 % (w/w) become too viscous to breakdown. But exploitation of horizontal bioreactors has proven effective in degrading pre-treated corn stover [84].

Many studies have also confronted the mass transfer issues, although more research is expected in this area, especially in pilot- and large-scale reactors. Studies suggest that utilizing a pre-mix in a fed-batch reactor with horizontal rotation can help combat this problem [85]. A prospective reactor can be developed with lower energy consumption and better mass transfer coefficients. In this regard, gas lift bioreactors and bubble column bioreactors have been put forth for consideration [82]. Researchers have explored the influence of varying pH [86] and alkali concentration [87] levels on the yield of reducing sugars in enzymatic hydrolysis and fermentation process. Besides cellulases, lignin degrading enzymes are also becoming a vital part of biofuel producing industries [88].

The unique standpoint of this study is the combination of experimental and computational methods of evaluation of cellulases for the production of bioethanol. Experimental validation of cellulase activity has been conducted extensively throughout literature. Their enzyme activity under different conditions is studied to optimize reaction conditions for biofuel production on a large scale. While wetlab techniques provide a realistic outlook towards functional aspects of cellulase bioconversion, they consume a lot of time, energy, capital and resources. In this regard, computational evaluation methods are a favorable alternative. Computational analyses of reaction parameters and conversion dynamics significantly shorten the time span necessary to study these in a reactor. They also provide a molecular-level understanding of the chemistry behind bioethanol production. As a novel strategy, a hybrid method has emerged, that combines the rapid screening of computational techniques and the conventional validation of laboratory procedures. This review will highlight both sides of the coin – experimental and computational study of cellulase activity for biofuel production from various sources.

## 2. Evaluation of cellulases

**Figure 2.**
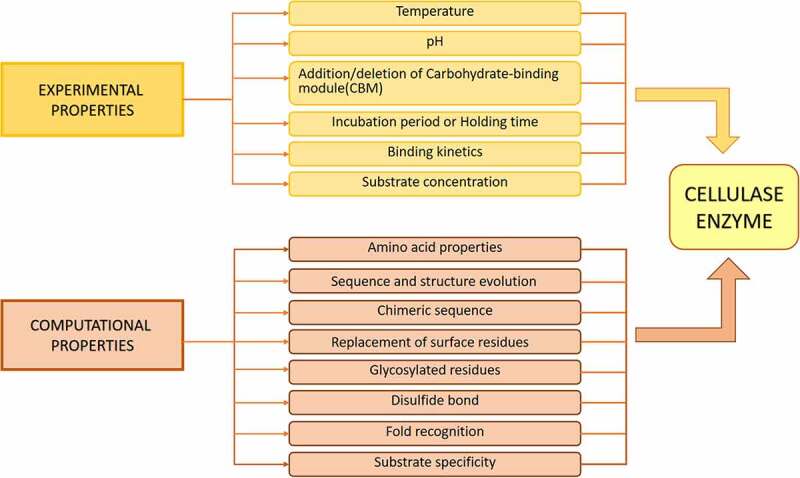
**Experimental and computational evaluation of cellulase properties**. There are multiple methods that can be used to evaluate various properties of cellulases.

There are multiple ways to evaluate cellulase properties. Figure 2 provides an overview of the current evaluation methods reported in the scientific literature. Unlike the experimental methods, computational methods such as sequence- and structure-based analyses use the scientific literatures information to extrapolate and predict cellulases’ various properties. This review is divided into two significant aspects of evaluation for cellulases: experimental and computational evaluations.

### 2.1. Experimental evaluation of cellulases

Physicochemical characteristics for cellulases’ characterization have been an active research area for decades [89]. Enzyme stability and activity at varying pH and increasing temperature are essential properties that need to be studied [90].

Researchers have explored three bacterial strains of *Cellulomonas* sp., *Bacillus* sp., and *Micrococcus* sp., for endoglucanase activity against coir fiber at different pH (ranging from 5 to 9) and temperature (ranging from 20 °C to 50 °C). Here, *Cellulomonas* sp., showed the highest activity at neutral pH and at 40 °C [91]. Fungal cellulase study of strain *Aspergillus niger* MS82 shows optimum enzyme activity at pH 4.0 at 35 °C [92].

A high-throughput method for evaluating temperature and pH dependence, simultaneously, of various enzymes using 96-well plate and a gradient PCR cycler has garnered attention because of its combined study criteria. The study demonstrated its applicability in the single enzyme (endoglucanase Cel8A from *Clostridium thermocellum*) and the commercially available complex enzyme mixture Celluclast® [93]. The above-discussed examples of studies providing detailed optimization criteria provide a starting step in designing laboratory experiments for yield enhancement.

The natural biomass complex components are cellulose, hemicelluloses (xyloglucan, xylan, and/or glucomannan), lignin, pectin, oil, fats, waxes, proteins, and various extractives. The synergy of the cocktail of enzymes acting on different components at the same time on the biomass is anticipated to give a high yield. For example, in 1999, a report talked about using a cocktail of pure cellulose-, xylan-, and mannan-degrading enzymes in birch and pine kraft pulp [94]. Later in 2008, a cocktail of xylanase and esterase on pretreated corn stover was suggested [95]. Saddler et al.’s extensive cocktail study with cellulases concluded that a good synergic interaction of endo-xylanases and xyloglucanases with cellulase improved biomass hydrolysis [96]. Similarly, xylanase and cellulase enzymes’ synergistic effect [97] and addition of accessory enzymes and cellulases are reported to enhance hydrolytic performance [98]. Østby et al. reported the interplay of enzymes and the relationship between enzymes used in a cocktail, their appropriate ratio, the impact of physicochemical conditions on enzyme activity, etc. [99].

Incubation time has also been reported as a factor for optimization. For example, extraction of cellulase from *A. niger* in varied carbon sources showed that the incubation period affects cellulase activity. The study reported a holding time of 10 min as an optimum time for expression of cellulase when wheat straw is used as a substrate [100]. It is also reported that the rate of product formation is not a linear reaction, and an increase in incubation time will not always increase activity and product formation. The optimum incubation time was identified as 24 hrs for bacterial cellulase in molasses [101].

The innovation in genome tailoring provides an opportunity to recreate desired improved potential strains with high enzymatic activity. Traditional chemical or physical mutagenesis approaches produced some improved strains; *Aspergillus* sp. XTG-4 [102], *T. viride* N879 [103], *Cellulomonas* sp. TSU-03 mutant M23 [104], and *Bacillus* sp. C1 mutant C1M26 [105]. However, mutants were incompetent in terms of cellulase production and activity, and were time-consuming; hence utilization of rational strategies to alter cellulase production is worth seeking. *Thermotoga maritima* Cel5A is an example of site-directed mutagenesis and CBM modification of endoglucanase, resulting in obtaining hyperthermostable enzymes [106].

Carbohydrate-binding modules (CBM) are essential components and increase the enzyme’s proximity to its substrate. Designing chimeric enzymes by modifying cellulase CBM to enhance their hydrolysis activity is a promising genetic engineering approach. Chimeric enzymes are synthesized by the fusion of the catalytic domain from one enzyme (of one species or organism) with that of the CBM from another species or organism’s enzyme. Recent studies have shown encouraging results of creating thermostable and thermotolerant chimeric enzymes [107] and increased substrate specificity [108–111].

Binding kinetics is another parameter that needs to be evaluated in cellulases. The study on Cel6B and Cel9A cellulases showed that while using the photobleaching method, an increase in temperature decreases the binding affinity while exhibiting partial reversibility in the presence of CBM [112]. Simultaneously, 45 °C temperature was not high enough to be detrimental to substrate binding for Cel5A; it may relate to the thermal stability of the protein also, determined by the protein fold, specifically, whether it is an (α/α)_8_, (α/β)_8_, or β-jelly roll fold [113]. According to a recent study, increasing the ratio of productive to non-productive binding sites promotes hydrolysis. To prevent hydrolysis slowdown during conversion, it is essential to maintain a high productive binding capacity [114].

Significance of enzymatic cocktails have been investigated in cellulase production and improvement [115,116]. Usage of enzymatic cocktails raised additional questions and challenges about interactions and interplay between enzymes that would be beneficial or detrimental, missing information on optimal enzyme ratios, and design of optimal genome tailoring routes to be deployed focusing on facilitated production. For instance, a recent study reported success in generating a “trigenic recombinant strain” of *Penicillium oxalicum* with improved cellulolytic activity through a combinatorial manipulation of three regulators, *clrB, bgl2*, and *creA*, in its regulatory pathway [115].

Another study focused on a systems biology approach and studied *T. reesei’* s 28 regulatory genes overexpression, to identify optimum conditions for enhanced cellulase production [117]. Interestingly, deletion of *ace3* gene was detrimental for cellulase production, which also significantly reduced xylanase production in the widely used cellulolytic organism *T. reesei* [117].

Experimental evaluation provides a qualitative view of reaction kinetics in real-time. Different organisms, enzymes and enzyme cocktails can be tested for LCB hydrolysis, at different physical and chemical conditions. Reaction parameters can be modified at any point in the process to observe changes in the system. It is also possible to detect, quantify, and characterize any inhibitors and/or toxic intermediates in the mixture. This is an especially useful step to perform in laboratory scale and pilot scale studies to avoid heavy losses in large-scale operations.

While experimental techniques have their place in the analyses of LCB breakdown, they come with their own set of downfalls. The main disadvantages of these techniques are the longer periods of time required to conduct the tests and the cost incurred thereof. Each reaction in the lineup of processes requires at least a few hours and culturing of microbes for microbial treatment of biomass demands anywhere between a few days to a few weeks time to grow to the required stage. It also compels extended periods of time for any mutation studies to lead to observable changes which prolongs the evaluation stage. Identifying high-yielding strains is a challenge in itself, and finding the right media, and optimum conditions for cellulase production are bigger obstacles.

The cost of running the machinery add up to a significant amount and also, cellulase enzymes are exorbitantly expensive. The cost is exponentially high for enzyme cocktails, modified and recombinant enzymes. Meeting these expenses in laboratories is difficult without adequate funding.

### 2.2. Future directions

Currently, enzyme cocktails, fungal cellulase production, and high-throughput screening variants seem to be the direction that researchers might want to take to discover close-to-ideal enzyme systems for biofuel production, particularly, biobutanol. Biobutanol is said to have better fuel properties than ethanol, and it is produced at higher efficiency by fungal systems. Moreover, utilizing fungal cellulases with other enzymes for enzyme cocktails is a smart choice to employ for faster, and economical fuel production. Lastly, high-throughput screening provides a means to select for higher yielding strains and enzymes in a fraction of the time, which is a bonus in these expeditious times.

### 2.3. Computational evaluation of cellulases

Yan and Wu reported predictors to identify optimum pH of cellulases in *Pyrococcus horikoshii* using a 20–1 feedforward backpropagation neural network [118] and also the prediction of optimal pH and temperature of cellulases using 20–2 feedforward backpropagation neural network [119]. BRENDA database provided the relevant properties of 20 amino acids used in the study for the cellulase enzyme class EC 3.2.1.4 [120–123].

Advances in computational methods can help predict cellulases’ physical and chemical properties, and information such as optimal pH ranges for the highest enzymatic activity. Piecing together this kind of essential information can guide future experimental studies. The sequence mutations and tertiary (i.e., three-dimensional) structure analyses of glycoside hydrolase 6 (GH6) family were performed to find the optimal pH for enzyme activity. The analyses showed that altering the properties of surface charge in GH6 family cellulases enhanced their activity by 62 % with respect to that of the wild type [124]. Another study conducted by Lugani et al. is the best example of utilization of *in silico* tools for the characterization of cellulase enzymes from different *Bacillus* species for their physicochemical characteristics, ancestral relationship, and structure determination at various levels [125].

The computational approach involves the usage of a repository of tools such as homology modeling [126], binding site identification [127], and molecular docking [128]. A study mainly consisted of 3D models (Modeler 9v9) of cellulase from *Acinetobacter* sp., prediction of substrates’ binding sites, and active site characterization based on the substrates’ docking studies [129]. Information of binding efficacy of enzyme with substrate might provide prospective substrates of choice for carbon and nitrogen sources. These docking studies revealed that cellulase has better affinity towards cellotetraose as a substrate for higher yield of ethanol among the selected substrates [129]. Tang et al. focused on the construction of mutants of 1,4-β-glucosidase with enhanced activity based on homology modeling, molecular docking, and the site-directed mutagenesis of target residues to modify spatial positions, steric hindrances, or hydrophilicity/hydrophobicity. The mutants created by site-directed mutagenesis were successfully expressed in the *Pichia pastoris* expression system and enhanced activity for the same mutants (pPICZαA-G235 M and pPICZαA-N347S) was verified. These type of findings guide alternative ways for improving the properties of 1,4-β-glucosidase [130].

Computational evolutionary and structural analyses of GH48 (classification according to the CAZy database) [131] enzymes encoded by horizontally transferred genes were performed to distinguish cellulase from non-cellulase proteins to reduce sample protein space upstream of a computational predictive pipeline. The essential structural element ω-loop on the surface of the GH48 enzyme significantly differentiates between cellulase and non-cellulase proteins [132,133]. The search for putative cellulases in metagenomic data was done using the highly conserved and rare amino acids of the ω-loop [134]. In another study, mutation and enzyme fusion analyses were used to improve the activity of hyperthermophilic β-1,4-endoglucanase (EGPh) from *Pyrococcus horikoshii*. Cysteines were mutated to disrupt the disulfide bonds, which increased the activity of mutated enzyme without the loss of thermostability. In the same study, fusion enzyme of EGPh with a chitin binding domain enhanced activity compared to wild type EGPh [135].

SCHEMA structure guided-recombination of three fungal class II cellobiohydrolases (CBH II cellulases) was used to construct a collection of highly thermostable CBH II chimeras. A sample set of 48 chimeric sequences out of a total of possible 6,561 sequences was chosen. Among 48, 23 were from a heterologous host, *Saccharomyces cerevisiae,* in their catalytically active form. Five chimeras showed a greater half-life thermal inactivation at 63 °C in comparison to the most stable parent. Twenty-five new CBH II sequences from thermophilic fungus *Humicola insolen*s were designed based on theoretical modeling of thermostabilities. Ten catalytically active chimeras out of 25 were more stable and active than those in the stable wild-type parent thermophilic strain *H. insolens*. A set of 15 sequences validated as CBH II thermostable enzymes showed high sequence diversity and hydrolyzed more cellulose than the parent enzyme [136].

Computational methods also identify the N-linked and O-linked glycosylated residues in the cellulase enzyme [137]. These residues affect stability, binding affinity, and catalytic efficiency. The N-linked glycosylated residues are primarily found in the glycoside hydrolases (GH) domains, whereas the O-linked glycosylated residues are mostly found in the linker regions between GH and CBM domains. Highly O-linked linker regions are protected from proteolytic degradation [137], and their identification is a part of high-precision protein engineering efforts.

Four broad methods of protein engineering have emerged over the decades. They are site-directed mutagenesis, directed evolution, computer-guided rational method, and semi-rational methods [138].

Site-directed mutagenesis involves targeting the active site of cellulases and hemicellulases by side chain modification [139,140]. In this strategy, enzymes can be modified to produce longer-chain alcohols, such as 3-Methyl-1-butanol, for their better conversion rates into biodiesel [141]. Alternatively, some enzymes have been shown to have preference for certain co-enzymes. But site-directed mutagenesis can reverse this preference to give better yields of ethanol [142]. Additional studies have been performed focusing on different components of the bioethanol production pathway to improve fuel yield.

In directed evolution methods, there is induction of random mutations, followed by extensive screening procedures to select for mutants with high bioethanol conversion rates [143]. These mutations lead to the generation of a large library of mutants, which are selected by high-throughput methods [144]. Computer-guided rational methods involve usage of computational techniques such as simulations, Quantum Mechanics calculations, Molecular Mechanics calculations, and docking studies [145]. These methods reduce the time required for analyzing enzyme properties, and screening thousands of compounds simultaneously. On the other hand, semi-rational methods are a combination of directed evolution and computational methods. Here, data from mutation studies is analyzed for designing enzyme active sites and scaffolds [146,147]. Combination of these methods provides a method to evaluate changes observed after directed evolution, and this information, along with structure–function relationship knowledge, is a smart way to formulate cellulase enzyme design.

The other protein engineering studies to improve cellulases toward enhanced activity include cellulose degradation, thermostability, pH stability, enhanced performance in non-conventional media, etc. They are well explained by Contreras et al. in a recently published article of 2020 [139]. The altering of transcription units on the genome by switching promoters or increasing copy numbers of cellulase genes, or creating fusion proteins are some of the approaches used in genetic tailoring[139,148–150].

Mathematical modeling and agent-based modeling/cellular automata have been used to model the kinetics of cellulose catalysis. An excellent review by Payne et al. describes these methods in detail [151]. There are additional methods available, such as molecular dynamics, constant-pH molecular dynamics, thermodynamic integration, quantum and molecular mechanics, and others that can successfully evaluate cellulases’ various properties. Among these methods, the sensitivity of the results depends on whether the cellulase is being analyzed at a fine-grained level (atomistic calculations) or a coarse-grained level (residue-level calculations). In some cases, it could be a mix of both. Detailed descriptions of these methods’ applications to evaluate cellulases are reviewed by Arora et al. [152].

Some of the computational methods used in evaluating cellulases described elaborately in literature include Constant pH Molecular Dynamics, Thermodynamic integration, Metadynamics, Continuum Molecular Dynamics, Monte Carlo methods, and Simulated Annealing. A brief description of each method is provided here as a guide for the readers. Since this short section does not do justice for these commonly used computational methods, we highly encourage the readers to refer to a large body of literature to learn more about these methods [153–156].

#### 2.3.1. Constant-pH molecular dynamics (CpHMD)

The method identifies the protonation states of titratable sites in a protein at a given pH. This method is helpful to understand the pH-dependent conformational changes that take place in a protein. Using this method, one can predict experimental pKa values and the dynamics induced at various pH values [157].

#### 2.3.2. Continuum-molecular dynamics

Multi-domain proteins such as cellulases are connected via a flexible linker region can leading inter-domain conformational changes. Longer MD simulations can identify nano to microsecond-time-scale changes, where the gradual macro-scale dynamical motions or continuum mechanics are ignored. The continuum-molecular dynamics method is an excellent alternative to generalize simulated tempering over a continuous temperature range to understand macroscale dynamics of the coupled dynamics of the catalytic subunit and CBM in cellulases [158].

#### 2.3.3. Simulated annealing

Simulated annealing (also known as generalized simulated annealing) is used to identify the most stable conformations of a protein, for example, in cases where the protein undergoes engineered mutations. When applied to a cellulase, the system is computationally heated to a high temperature then it is gradually cooled to reach the lowest energy functional states of the enzyme [159].

#### 2.3.4. Quantum and molecular mechanics

Quantum mechanical (QM) approaches can model accurate electronic rearrangements of active site atoms. However, they are computationally expensive [160]. Alternatively, molecular mechanics (MM) methods use more approximated force fields that are less accurate than QM, but they are faster and therefore computationally cheaper. The hybrid QM/MM methods are an option to overcome the limitations of a full quantum mechanical or a full molecular mechanics modeling, where the system is treated in part at the level of quantum chemistry (QM), retaining the computationally cheaper force field (MM) for the larger part.

## 2.4. Evaluation of cellulases for glucose yield in a hybrid production process.

Although it is slightly beyond our review scope, given that pretreatment during the biofuel production process is one of the most critical steps that can influence cellulase enzyme efficiency during industrial production (Table 1). Ishiguro and Endo [161] considered the possibility of a hybrid processing approach to increase glucose yield. Two well-known pretreatment methods, the alkali method, popular in bioethanol production and the hydrothermal method frequently used in paper and pulp industries, were combined.

**Figure 3.**
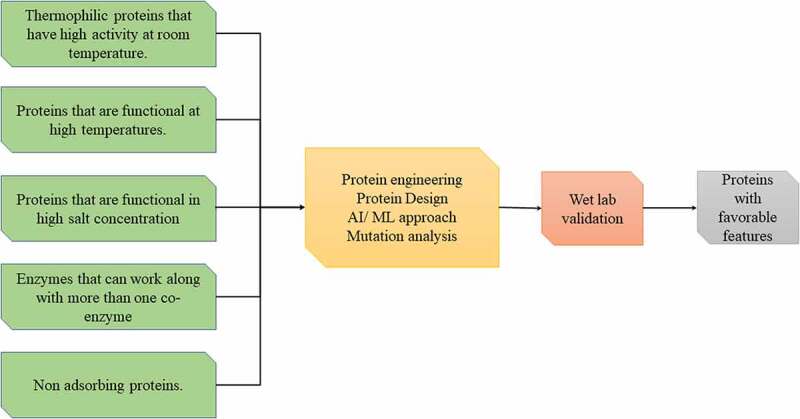
**Methods to engineer proteins with favorable or desired qualities/characteristics**. A schematic representation of futuristic engineering proteins with favorable qualities using machine learning and/or artificial intelligence approaches.

Figure 3 recapitulates the approach proposed by Ishiguro and Endo, where the first prerequisite step was reducing the size of hardwood biomass of Eucalyptus to 3 mm to decrease the tenacious nature of the wood, followed by the applications of different concentrations of sodium hydroxide (NaOH) and at various high temperatures in a reactor. The samples are then wet ball-milled for four hours. The required alkali fraction, for dissolving the lignin content, is removed by thorough washing before the next step of lyophilization, which was performed over a week’s time. The enzymatic saccharification step was performed for 48 hours, and the glucose yield was measured. The findings indicated increase in glucose yield by 55 % at 20 % sodium hydroxide solution at 170 °C. The hydrothermal process makes the recalcitrant cellulose microfibrils amenable for further digestion by reducing the particle size and converting it into carbonaceous materials, thereby providing a promising proof-of-concept for translating the process at an industrial scale.

## 3. Conclusion

Non-judicious consumption of conventional fossil fuel mandates a shift to renewable and sustainable sources of energy. The drive for biofuels primarily originates from the desire to reduce greenhouse emission against the deleterious effects of climate change. Still, economic assessment of biofuel supply chain and production as well as the trade-off of using traditional fuels facilitate its adoption as a fuel alternative, and possibly even future replacement of conventional fuels.

Evaluation of enzymes is an essential step in any biochemical process. The activity of the catalyst and its turnover rate determine the cost, time, and yield of the valuable end-product. In bioethanol, the yield of glucose at the end of the pretreatment and fermentation/saccharification process indicates the ease with which industrial and commercial demands can be met. Over the years, ample experimental and computational methods have been standardized for the physico-chemical analysis and exploration of biological properties of cellulases, the bioethanol industry’spotential catalyst.

Recently, a new chemocatalyst approach reported cellulose’s direct conversion to ethanol using a chemocatalyst consisting of molybdenum and platinum [162]. It involves a one-step route of the tandem reaction, cellulose conversion to ethylene glycol and then to ethanol in the same reaction setup, aptly called ‘one-pot production’. The advantages of the chemocatalytic process make it a promising sustainable alternative to the current bioprocess; translating this approach into large-scale ethanol production in a real-world scenario can be a new research area for investigators. The interdisciplinary research and global trends coupled with heterogeneity of supply and demand systems, and economic analyses create a highly complex set of challenges. The scientific and technological aspects need attention to give rise to developing potential methods, stable and efficient enzymes, minimizing the steps of processing, and ultimately cost-effectiveness. Researchers also need to find an answer to the economical challenges, such as the cost of corn production, trade-off of using corn as a biofuel precursor instead of food or feed, the ultimate cost of building and operating plants of biofuel production, and the relative overall cost of biofuel end-product against conventional fuels (e.g., oil).

In this review, we highlight the numerous methods used to evaluate various properties of cellulases, experimentally and computationally. While experimental evaluation is ideal, there are instances where computational evaluation has provided new biological insights and saved time, thus having an economic advantage. The active research area of using hybrid methods that combines more than one pretreatment process is gaining researchers’ attention [161]. There are areas yet to be explored, such as integrating computational and experimental outcomes, creating standard testing and validation guidelines, and using machine learning and artificial intelligence methods to expand our understanding of biofuel enzymes to develop more optimized industrial processes.

The industry of biofuel production has picked up pace in the last two decades in view of the impending complete exhaustion of fossil fuels, and also the need for more sustainable, greener alternatives that deal with the enormous amount of biomass waste generated. With advancements in cellulase production technology, protein engineering to enhance cellulase activity, and methods to analyze production parameters and strategies, many milestones have been reached, and yet several more remain. Prospects in this arena are aplenty.

For example, a recent topic of interest has been the production of thermostable cellulolytic enzymes, which can be beneficial in many ways, such as higher rates of bioconversion, minimized contamination by microorganisms, and abated costs required for plant cooling [163]. Butanol seems to be the alcohol of choice as per research in the last two decades [164]. Although many Clostridia are known to be excellent producers of butanol, and several mutants have been created to maximize production, their full-fledged large-scale production is still underway since that necessitates additional studies and optimization.

This is where rational computational methods and hybrid techniques come into the picture: to estimate reaction conditions, predict unfavorable process parameters, and analyze potential properties by simulations and docking studies. According to predictions, systems biology studies are next in the pipeline to help conceptualize, design, and implement biofuel production strategies.
